# Sulforaphane Ameliorates the Severity of Psoriasis and SLE by Modulating Effector Cells and Reducing Oxidative Stress

**DOI:** 10.3389/fphar.2022.805508

**Published:** 2022-01-21

**Authors:** Pei Du, Wenqian Zhang, Haobo Cui, Wei He, Shuang Lu, Sujie Jia, Ming Zhao

**Affiliations:** ^1^ Department of Dermatology, Second Xiangya Hospital, Central South University, Changsha, China; ^2^ Hunan Key Laboratory of Medical Epigenomics, Second Xiangya Hospital, Central South University, Changsha, China; ^3^ Research Unit of Key Technologies of Diagnosis and Treatment for Immune-related Skin Diseases, Chinese Academy of Medical Sciences, Changsha, China; ^4^ Clinical Medical Research Center of Major Skin Diseases and Skin Health of Hunan Province, Changsha, China; ^5^ Department of Pharmacy, The Third Xiangya Hospital, Central South University, Changsha, China

**Keywords:** antioxidant effect, sulforaphane, natural compounds, psoriasis, SLE

## Abstract

**Background:** Sulforaphane, which is found in cruciferous vegetables, has been reported to have anti-inflammatory, antioxidant, and antitumour activities. However, whether sulforaphane has therapeutic effects on inflammatory or autoimmune skin diseases, including psoriasis and systemic lupus erythematosus (SLE), is unclear.

**Methods:** The therapeutic effects of sulforaphane were analyzed in Imiquimod (IMQ)-induced psoriasis-like mice and lupus-prone MRL/lpr mice. In IMQ-induced psoriasis-like mice treated with sulforaphane (55.3 and 110.6 μmol/kg) or vehicle control, the pathological phenotypes were assessed by the psoriasis area and severity index (PASI) score, haematoxylin-eosin staining (H&E) and quantifying of acanthosis and dermal inflammatory cell infiltration. The proportions of T cell subsets in draining lymph nodes (dLNs) and spleens were examined by flow cytometry. In MRL/lpr mice treated with sulforaphane (82.9 μmol/kg) or vehicle control, mortality and proteinuria were observed, and the glomerular pathology was examined by H&E staining. C3 and IgG depositions in kidney sections were examined by immunofluorescence staining. The proportions of plasma cells, follicular helper T (Tfh) cells, neutrophils and dendritic cells in the dLNs and spleens were examined by flow cytometry. Finally, we examined the Malondialdehyde (MDA) concentration by thiobarbituric acid reactive substance assay and the expression of *Prdx1*, *Nqo1*, *Hmox1*, and *Gss* by reverse transcription-quantitative polymerase chain reaction (RT-qPCR).

**Results:** Sulforaphane ameliorated the skin lesions in IMQ-induced psoriasis-like mice and the renal damage in lupus-prone MRL/lpr mice. In IMQ-induced psoriasis-like mice, sulforaphane reduced the proportions of Th1 and Th17 cells and increased the expression of antioxidant gene *Prdx1*. In lupus-prone MRL/lpr mice, sulforaphane increased the lifespan and the expression of *Prdx1*, and decreased the proportions of plasma cells, Tfh cells, neutrophils, and dendritic cells in the dLNs and spleens and the concentration of MDA.

**Conclusion:** Sulforaphane has significant therapeutic effects on IMQ-induced psoriasis-like mice and lupus-like MRL/Lpr mice by reducing inflammatory and autoimmune-related cells and oxidative stress. These findings provide new evidence for developing natural products to treat inflammatory and autoimmune diseases.

## Introduction

**FIGURE 1:**
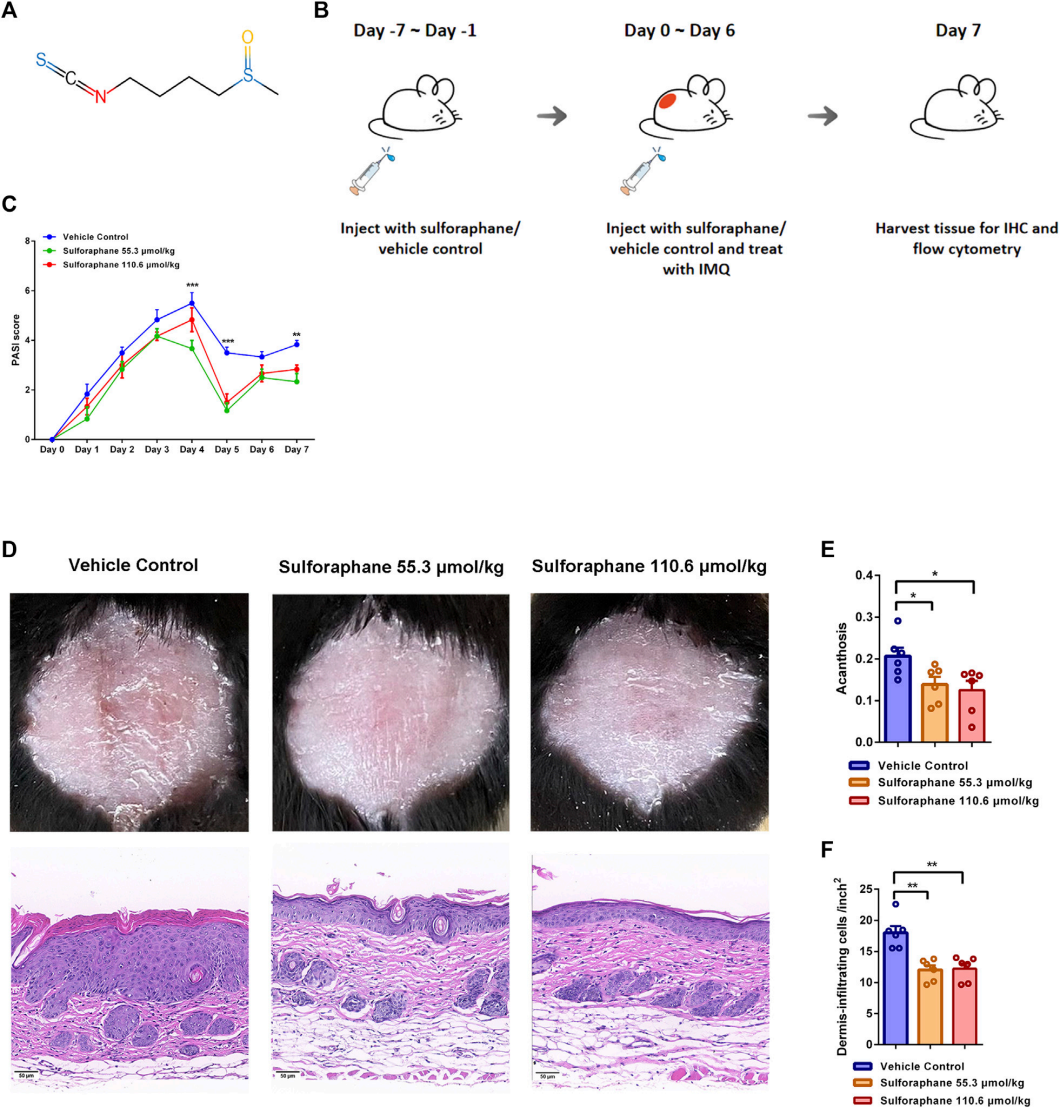
Effects of sulforaphane on IMQ-induced psoriasis-like mouse model. **(A)** The structure of sulforaphane. **(B)** Schematic diagram of sulforaphane administration (55.3 and 110.6 μmol/kg, i.p.) or vehicle control for 7 consecutive days before IMQ treatment, followed by daily treatment with 78 mg of IMQ cream (5%) on the shaved back and injection with sulforaphane or vehicle control for 7 consecutive days. **(C)** PASI scores of mice in each group (n = 6). Compared with vehicle control, PASI scores were significantly decreased in mice treated with sulforaphane (55.3 μmol/kg) on Day 4, 5, and 7 and in mice treated with sulforaphane (110.6 μmol/kg) on Day 5. **(D)** Appearance and H&E staining of lesional skin from mice that were administrated with sulforaphane or vehicle control. Scale bars: 50 µm. **(E, F)** Quantitation of acanthosis and dermal inflammatory cell infiltration was performed in each group. The data represent the mean ± SEM. *p < 0.05, **p < 0.01, ***p < 0.001.

Recently, a growing number of studies have shown that natural compounds extracted from plants have satisfying effects for the treatment of human diseases. For example, artemisinin, which is extracted from *Artemisia annua L.*, is the most widely used natural compound against malaria and plays a vital role in the treatment of malaria worldwide (Ma et al., 2020). Cruciferous vegetables have been considered sanatory, and can reduce the risk of chronic diseases (Raiola et al., 2017). The bioactive compounds that have been reported are ascorbic acid, phenolics, carotenoids, and glucosinolates (Peluso and Palmery, 2014). Sulforaphane, also known as isothiocyanate 1-isothiocyanato-(4R)-(methylsulfinyl) butane (Figure 1A), is a kind of isothiocyanate that is a hydrolysate of glucosinolates produced by the enzyme myrosinase in cruciferous vegetables (Raiola et al., 2017). Sulforaphane has been reported to exhibit the anti-inflammatory, antioxidant, and antitumour activities. Sulforaphane exerted an anti-inflammatory effect by regulating MAPK signaling in lipopolysaccharide (LPS)-induced microglia (Subedi et al., 2019). Furthermore, sulforaphane exerts therapeutic effects on diabetic nephropathy by upregulating nuclear factor-like 2 (Nrf2) antioxidant signaling (Li et al., 2020). In addition, the antitumour effects of sulforaphane in clinical therapy range from the attenuation of DNA damage to regulation of the cell cycle by activating the transcription factor Nrf2 (Russo et al., 2018). However, whether sulforaphane has therapeutic effects on inflammatory or autoimmune skin diseases, including psoriasis and systemic lupus erythematosus (SLE), is unclear.

Psoriasis is a chronic inflammatory immune-related skin disease characterized by erythema, scales, epidermal hyperplasia, and infiltration of inflammatory cells (Beek and van Reede, 1977). The release of proinflammatory mediators and cytokines has been identified in the skin and systemically (Nickoloff et al., 2007). Dysfunction of T lymphocytes, especially Th1 and Th17 subtypes, causes the overexpression of interferon-γ, interleukins, and tumor necrosis factor (Wu et al., 2018). Notably, oxidative stress is involved in the pathogenesis of psoriasis. A number of oxidative stress-related markers are increased in the plasma or serum of psoriatic patients, such as ischaemia-modified albumin (Kirmit et al., 2020), catalase (Kirmit et al., 2020), malondialdehyde (Kadam et al., 2010), and nitric oxide (Gabr and Al-Ghadir, 2012). Furthermore, the elevated levels of reactive oxygen species (ROS) are caused by active oxidative stress and initially trigger the abnormal activation of Th1 and Th17 cells (Lai et al., 2018). Moreover, ROS act as secondary messengers that induce activation of the MAPK, NF-κB, and JAK-STAT signaling pathways, amplifying the inflammatory response (Lin and Huang, 2016). Therefore, targeting oxidative stress may be a therapeutic strategy for psoriasis. IMQ-induced psoriasis-like skin inflammation is characterized by acanthosis, parakeratosis, and infiltration of inflammatory cells, which resemble human psoriasis. The mouse model uses the daily topical application of imiquimod (IMQ), a TLR7/8 ligand, to induce the inflammation of skin (Singh et al., 2019). The model is used to investigate the molecular and cellular pathogenesis of psoriasis in preclinical studies, as well as in the evaluation of potential therapies.

SLE is a systemic autoimmune disease that affects multiple organs, including the skin, kidney, and joints. The pathogenesis of SLE has not yet been clarified. The loss of immunologic tolerance causes the aberrant activation of autoreactive T cells and B cells, inducing massive autoantibodies production. Eventually, complement activation and the deposition of immune complexes lead to organ damage. In SLE, neutrophil extracellular traps released by neutrophils enhance the activation of inflammasomes and accumulate due to impaired degradation, causing increased anti-dsDNA production (Yu and Su, 2013). Dysfunction of dendritic cells is associated with the development of SLE. Inappropriate antigen presentation by dendritic cells promotes the loss of immunologic tolerance, accelerating the progression of SLE (Tsokos et al., 2016). Oxidative stress is closely related to the progression of SLE. Excessive production and impaired clearance of ROS induce dysfunction in T cells (Perl, 2013). In addition, oxidative modification of self-antigens triggers autoimmunity, leading to the acceleration of SLE (Perl, 2013). Studies have demonstrated that blocking the antioxidant signaling pathway reduces lifespan and increases autoantibodies, aggravating renal damage (Yoh et al., 2001). The elevated oxidative stress correlates with disease activity and organ damage. Thus, antioxidant therapy might provide an important strategy to ameliorate SLE. MRL/MpJ-Fas^lpr/lpr^ (MRL/lpr) mice are the defect of *Fas* gene on MRL/MpJ background, which fail to eliminate autoreactive lymphocytes, inducing spontaneous autoimmune phenotypes. The clinical manifestations of MRL/lpr mice resemble human SLE, including skin lesion, glomerulonephritis, arthritis, circulating anti-nuclear antibodies (ANA), and depositions of immune complexes and complements (Watanabe-Fukunaga et al., 1992). MRL/lpr mice are used to study molecular and cellular pathogenesis of SLE in preclinical studies and evaluation of potential therapies.

To clarify the effects of sulforaphane on psoriasis, we studied the differences in skin inflammation and changes in Th subtypes in IMQ-induced psoriasis-like mice treated with sulforaphane or vehicle control Our findings suggested that sulforaphane reduced the percentages of Th1 and Th17 cells to inhibit skin inflammation. Moreover, we investigated the potential therapeutic effect of sulforaphane on SLE. Sulforaphane increased the lifespan and reduced the renal damage in lupus-like mice and decreased the percentages of plasma cells, Tfh cells, neutrophils, and dendritic cells. These results indicated that sulforaphane is a natural compound with therapeutic effects on skin inflammation and autoimmune diseases.

## Materials and Methods

### Mice

Female C57BL/6 mice aged 6–8 weeks were purchased from Slack and were used for the IMQ-induced psoriasis-like model. Female MRL/lpr mice aged 8 weeks were purchased from Sibeifu Experimental Animal Co. Ltd. All mice were housed under specific pathogen-free conditions at a controlled temperature of 20–25°C and 35–75% humidity with a 12-h light/dark cycle and were provided sterile food and water ad libitum. Animal experiments were performed according to the Institutional Animal Care Guidelines and were approved by the Animal Care Committee of Second Xiangya Hospital of Central South University.

### Sulforaphane Administration to IMQ-Induced Psoriasis-Like Mice and MRL/Lpr Mice

Sulforaphane is a single compound purchased from APExBIO (Cat. C4733). Female C57BL/6 mice (6–8 weeks of age) were divided into 3 groups. Before being treated with IMQ, all mice were injected daily with sulforaphane (55.3 and 110.6 μmol/kg, intraperitoneal injection) or vehicle control (i.p.) for 1 week. Then, all mice were administered 78 mg of IMQ cream (5%) (Sichuan Med-shine Pharmaceutical) daily on their shaved backs and continually injected with sulforaphane (55.3 and 110.6 μmol/kg, i.p.) or vehicle control (i.p.) daily for 7 consecutive days. The skin, dLNs, and spleens of IMQ-induced psoriasis-like mice were obtained for further detection. After 6 weeks, female MRL/lpr mice (14 weeks of age) were divided into 2 groups. All mice were injected with sulforaphane (82.9 μmol/kg i.p.) or vehicle control (i.p.) daily for 27 consecutive days. The proteinuria was detected once a week to evaluate injury of kidney. The kidneys, dLNs, and spleens of MRL/lpr mice were obtained for further detection. Cell suspensions of spleens and dLNs were obtained by passing the tissues through a 70 µm strainer for flow cytometry analysis.

### Evaluation of the Severity of Skin Inflammation in a Psoriasis-Like Mouse Model

The severity of skin inflammation in each mouse was scored once per day according to the criteria. The severity of skin inflammation was assessed according to 3 symptoms: erythema, scaling, and thickening. The score of each symptom ranged from 0 to 4 as follows: 0, none; 1, slight; 2, moderate; 3, marked; and 4, very marked. The total score was the sum of the 3 index scores (score 0–12).

### Histological Analysis

Mouse skin and kidney tissues were fixed in 4% paraformaldehyde for 24 h at room temperature and embedded in paraffin. Sections (6 μm) were stained with haematoxylin and eosin (H&E). Acanthosis and the number of dermis-infiltrating cells were assessed as histological features of skin inflammation. The histological analysis of skin was performed as reported (Wu et al., 2018). The relative area of the epidermis was calculated for each mouse. The number of dermis-infiltrating cells in each section was calculated from three random fields of view at ×20 magnification.

### Flow Cytometry

The following monoclonal antibodies were used for flow cytometric analysis of immune cells: anti-mouse CD4 (BD Pharmingen, Cat. 553088), anti-mouse IFN-γ (BD Pharmingen, Cat. 560660), anti-mouse IL-4 (BD Pharmingen, Cat. 560699), IL-17a (BD Pharmingen, Cat. 561020), CD8 (Biolegend, Cat. 100744), B220 (BD Pharmingen, Cat. 563894), CD11c (BD Pharmingen, Cat. 746392), CXCR5 (Biolegend, Cat. 560617), CD138 (BD Pharmingen, Cat. 568626), IgD (BD Pharmingen, Cat. 553510), CD19 (Biolegend, Cat. 115530), CD45 (BD Pharmingen, Cat. 560510), CD11b (BD Pharmingen, Cat. 564454), and Gr-1 (Biolegend, Cat. 108410). For cytokine analysis, cells were stimulated in RPMI 1640 medium (Gibco) supplemented with 10% fetal bovine serum (FBS, HyClone), PMA, ionomycin, and GolgiPlug (BD Biosciences, Cat. 550583) at 37°C and 5% CO_2_ for 6 h. For intracellular staining, cells were fixed and permeabilized with a Human Foxp3 Buffer Set (BD Biosciences, Cat. 562574) or Cytofix/Cytoperm (BD Biosciences, 554722) according to the manufacturer’s instructions.

### RNA Isolation, Reverse Transcription and Real-Time PCR

Total RNA was extracted from cells or tissues with TRIzol reagent (Invitrogen) and reverse transcribed with the PrimeScript RT reagent kit with gDNA Eraser (TaKaRa Biotech Co.) according to the manufacturer’s instructions. qPCR was performed with SYBR Premix Ex Taq II (Tli RNaseH Plus) (TaKaRa Biotech Co.) in a LightCycler 96 (Roche) thermocycler. Relative mRNA expression was calculated using the 2^−ΔCt^ (ΔCt = Ct_target gene_−Ct_housekeeping gene_) method in the experimental group compared with the control group. The primer sequences are shown in Supplementary Table 1.

### Immunofluorescence Staining

Frozen sections of mouse kidneys were stained with a rat anti-C3 antibodies (11H9, Abcam) and Cy3-conjugated goat anti-rat IgG (GB21302, Servicebio) overnight at 4°C. The same procedure was performed for mouse IgG and direct immunofluorescence analysis with Alexa Fluor 488-conjugated goat anti–mouse IgG (150113, Abcam) overnight at 4°C.

### Assay of Malondialdehyde Concentration

The MDA concentrations in skin of IMQ-induced psoriasis-like mice and spleens and dLNs of MRL/lpr mice were detected with the Micro Malondialdehyde Assay Kit (Beijing Solarbio Science and Technology Co.) following the manufacturer’s instructions.

### Statistics

The data was shown as the mean ± standard error of the mean (SEM). Statistical analysis was performed with GraphPad Prism. A two-tailed *t* test was used for statistical analysis. When the data were normally distributed, ANOVA and Dunnett’s multiple comparisons test were used for grouped data analysis. When the data were not normally distributed or displayed unequal variances between two groups, we used the two-tailed Mann-Whitney U test for statistical analysis. When the data were normally distributed or displayed unequal variances between two groups, we used the unpaired Student’s *t* test. The log-rank (Mantel-Cox) test was used for survival analysis. *p* < 0.05 was considered statistically significant. Sample sizes for all data shown can be found in the figure legends.

## Results

### Sulforaphane Ameliorates the Skin Lesions in IMQ-Induced Psoriasis-Like Mice

We investigated whether sulforaphane exerted the anti-inflammatory effect on IMQ-induced psoriasis-like mice. Previous study has shown that the pathological phenotypes in 2,4-dinitrochlorobenzene-induced atopic dermatitis-like mice were significantly improved when sulforaphane was used three times a week for 3 weeks with a concentration gradient of 13.8, 27.6, and 55.3 μmol/kg (Wu et al., 2019). As a short course of IMQ-induced psoriasis-like mice model, skin lesions healed themselves after 7 days of induction, we chose two relatively high doses (55.3 and 110.6 μmol/kg) of sulforaphane to treat IMQ-induced mice in this study. Sulforaphane or vehicle control was injected daily (i.p.) in C57BL/6 mice for 7 consecutive days before IMQ stimulation. Then, all mice were treated with 78 mg of IMQ cream (5%) on their shaved backs and injected with sulforaphane or vehicle control (i.p.) daily for 7 consecutive days. The experimental design is shown in Figure 1B. As expected, the PASI scores, which indicate the severity of skin lesions in mice, were reduced in the sulforaphane treatment group compared with the vehicle control group (Figure 1C). Gross appearance and H&E staining indicated that skin lesions were significantly alleviated in mice treated with sulforaphane (Figure 1D). In addition, acanthosis and dermal inflammatory cell infiltration were significantly decreased after sulforaphane treatment (Figures 1E,F).

### Sulforaphane Reduced the Percentages of Th1 Cells and Th17 Cells in IMQ-Induced Psoriasis-Like Mice

**FIGURE 2:**
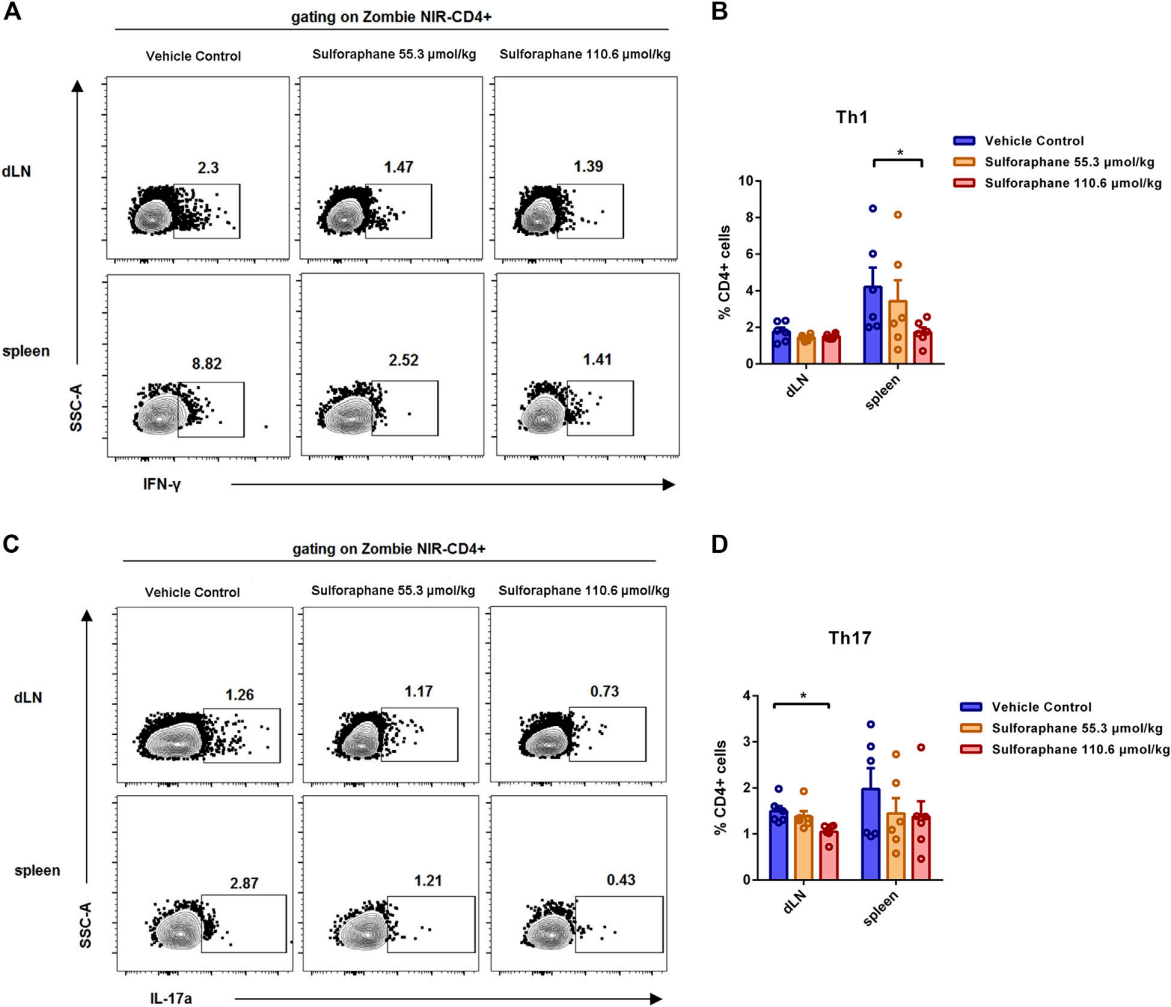
Sulforaphane reduced the proportions of Th1 cells and Th17 cells in IMQ-induced psoriasis-like mice. **(A)** Representative flow cytometric analysis of Th1 cells in the dLNs and spleens from IMQ-induced psoriasis-like mice treated with sulforaphane or vehicle control (n = 6). **(B)** Statistical analysis of **(A)**. **(C)** Representative flow cytometric analysis of Th17 cells in the dLNs and spleens from IMQ-induced psoriasis-like mice treated with sulforaphane or vehicle control. **(D)** Statistical analysis of **(C)**. The data represent the mean ± SEM. *p < 0.05.

Increasing evidence has demonstrated that inflammatory T cells such as Th1 and Th17 cells in skin lesions play important roles in the pathogenesis of psoriasis (Kagami et al., 2010; Jiang et al., 2020, Diani et al., 2015). Therefore, we sought to evaluate whether the amelioration of skin inflammation was attributed to the decreased differentiation of inflammatory T cells after sulforaphane administration. We examined the percentages of Th1, Th2, and Th17 cells in dLNs and spleens from IMQ-induced psoriasis-like mice. The gating strategy is shown in Supplementary Figure S1. Notably, we found that the percentage of Th1 cells in the spleens was significantly reduced in the sulforaphane-treated (110.6 μmol/kg) group (Figures 2A,B). The percentage of Th17 cells in the dLN was significantly reduced in the sulforaphane-treated (110.6 μmol/kg) group (Figures 2C,D). The percentage of Th17 cells in the spleens was decreased in the sulforaphane-treated groups, but the difference was not significant (Figures 2C,D).

### Sulforaphane Alleviates Renal Damage in Lupus-Prone MRL/Lpr Mice

**FIGURE 3:**
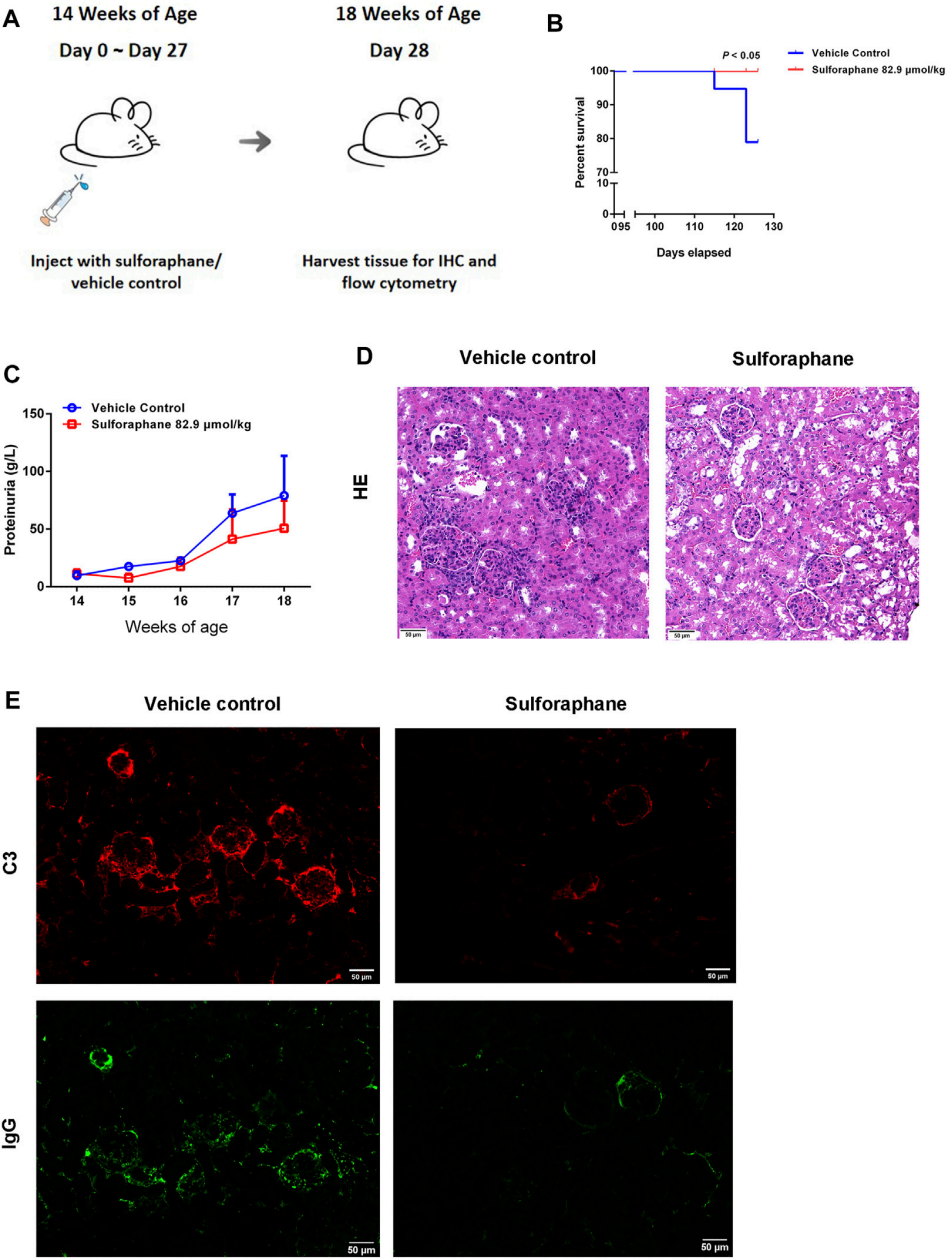
Sulforaphane alleviates renal damage in lupus-prone MRL/lpr mice. **(A)** Schematic diagram of the administration of sulforaphane (82.9 μmol/kg) or vehicle control for 27 consecutive days in MRL/lpr mice at the age of 14 weeks (n = 5–6). **(B)** The lifespan of MRL/lpr mice treated with sulforaphane or vehicle control. **(C)** The changes of urine protein levels in MRL/lpr mice treated with sulforaphane or vehicle control. **(D)** Renal pathology was examined by immunohistochemistry of haematoxylin-eosin staining (H&E) in MRL/lpr mice treated with sulforaphane or vehicle control. Scale bars: 50 µm. **(E)** C3 and IgG deposition in kidney sections was examined by immunofluorescence staining in MRL/lpr mice treated with sulforaphane or vehicle control. Scale bars: 50 µm.

Because of the anti-inflammatory effect of sulforaphane on IMQ-induced psoriasis-like mice, we hypothesized that sulforaphane was involved in regulating the autoimmune lymphocytes to alleviate kidney injury in lupus-prone MRL/lpr mice. Since both concentrations (55.3 and 110.6 μmol/kg) significantly improved skin lesions of IMQ-induced psoriasis-like mice, with no side effects, we chose a medium dose of sulforaphane (82.9 μmol/kg i.p.) to treat MRL/lpr mice for 27 consecutive days. The administration of sulforaphane significantly reduced the mortality of MRL/lpr mice (Figure 3B). Proteinuria was decreased in the sulforaphane-treated group compared with the vehicle control group (Figure 3C). In addition, sulforaphane-treated MRL/lpr mice had reduced glomerular enlargement as shown by H&E staining (Figure 3D). Immunofluorescent staining indicated that the deposition of C3 and IgG was dramatically decreased in the sulforaphane-treated group (Figure 3E). These results indicate that sulforaphane alleviates the autoimmune responses in the kidneys of lupus-prone MRL/lpr mice.

### Sulforaphane Reduces the Proportions of Plasma Cells, Tfh Cells, Neutrophils, and Dendritic Cells in MRL/Lpr Mice

**FIGURE 4:**
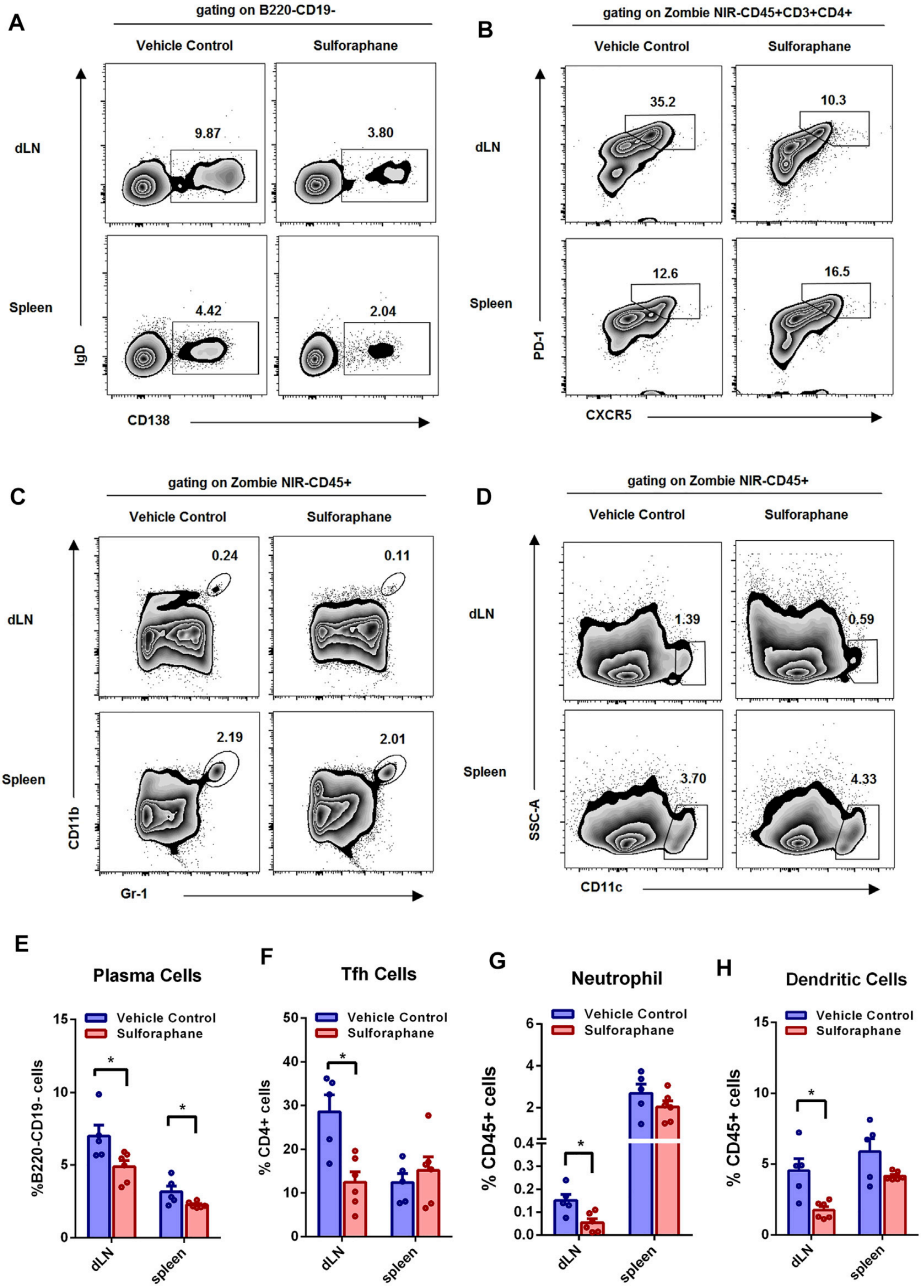
Sulforaphane reduced the percentages of plasma cells, Tfh cells, neutrophils, and dendritic cells in lupus-prone MRL/lpr mice (n = 6). **(A–D)** Representative flow cytometric analysis of plasma cells **(A)**, Tfh cells **(B)**, neutrophils **(C)** and dendritic cells **(D)** in the dLNs and spleens from MRL/lpr mice treated with sulforaphane or vehicle control. **(E–H)** Statistical analysis of **(A–D)**. The data represent the mean ± SEM. *p < 0.05.

Accumulating evidence has demonstrated that the aberrant responses of the innate and adaptive immune systems are involved in the pathogenesis of lupus. Next, we sought to explore whether sulforaphane influenced the activation and differentiation of B cells, Tfh cells, and innate immune cells in the spleens and dLNs of a lupus-like mouse model. The gating strategy is shown in Supplementary Figures S2, S3. We found that the percentages of plasma cells in the dLNs and spleens were significantly reduced in the sulforaphane-treated group (Figures 4A,E). And the proportions of Tfh cells, neutrophils, and dendritic cells were significantly decreased in the dLNs of sulforaphane-treated mice compared with vehicle control mice (Figures 4B–D,F–H). These results indicate that sulforaphane alleviates autoimmune response by inhibiting the activation or differentiation of plasma cells, Tfh cells, neutrophils, and dendritic cells in MRL/lpr mice.

### Sulforaphane Upregulates the Expression of Antioxidant Gene *Prdx1* to Reduce Oxidative Stress

**FIGURE 5:**
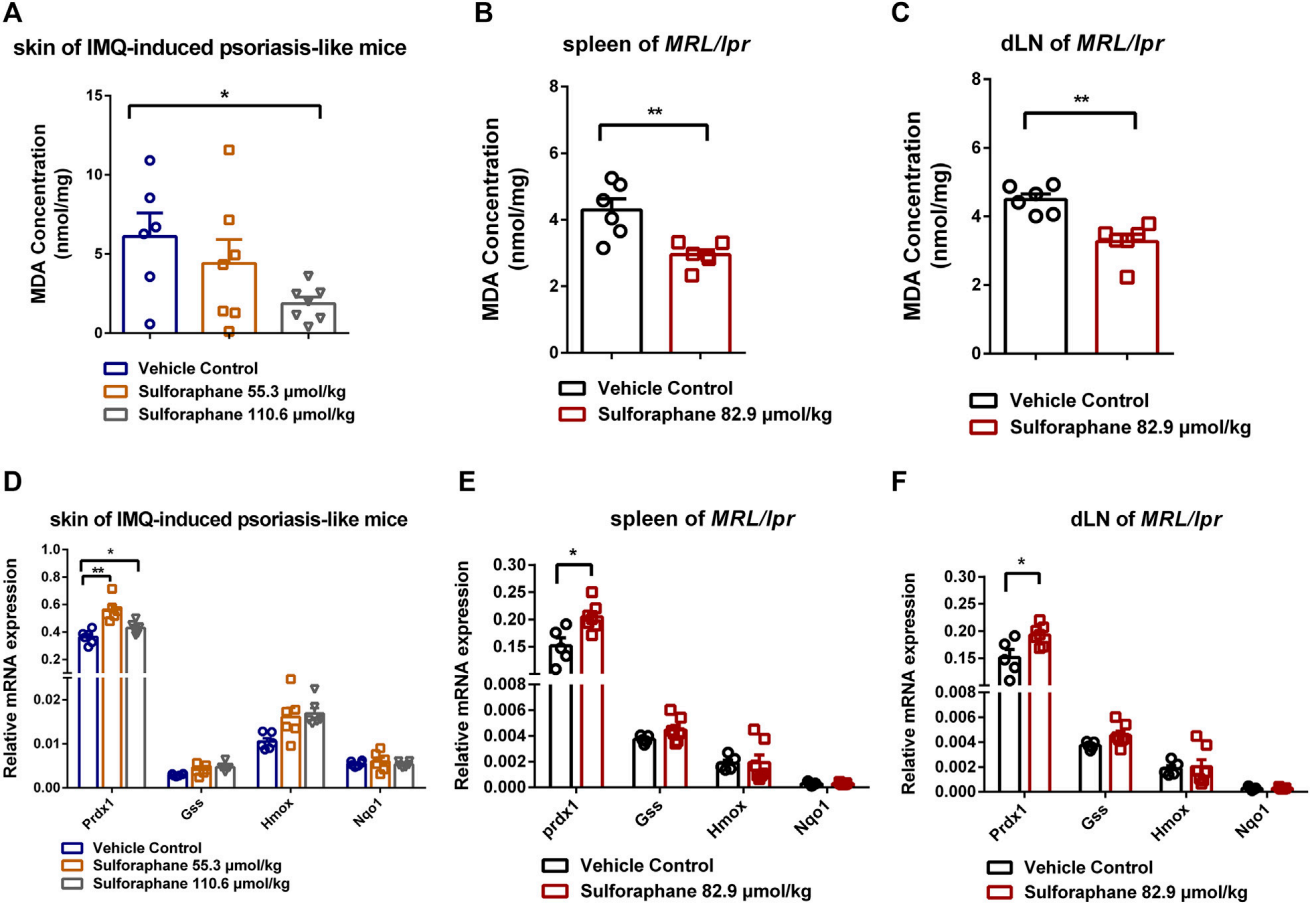
Sulforaphane upregulated the antioxidant gene *Prdx1* expression to reduce the level of oxidative stress. **(A)** The concentrations of MDA in skin of IMQ-induced psoriasis-like mice treated with sulforaphane or vehicle control (n = 5–6). **(B, C)** The concentrations of MDA in the spleens **(B)** and dLNs **(C)** of MRL/lpr mice treated with sulforaphane or vehicle control (n = 5–6). **(D)** The relative mRNA expression of *Prdx1*, *Gss*, *Hmox1*, and *Nqo1* in skin of IMQ-induced psoriasis-like mice treated with sulforaphane or vehicle control (n = 5–6). **(E, F)** The relative mRNA expression of *Prdx1*, *Gss*, *Hmox1*, and *Nqo1* in the spleens **(E)** and dLNs **(F)** of MRL/lpr mice treated with sulforaphane or vehicle control (n = 5–6). The data represent the mean ± SEM. *p < 0.05, **p < 0.01.

We next investigated the MDA concentrations in skin of IMQ-induced psoriasis-like mice and spleens and dLNs of MRL/lpr mice to indicate the level of oxidative stress between the sulforaphane-treated group and vehicle control-treated group. The treatment of sulforaphane reduced the concentrations of MDA in the skin of IMQ-induced psoriasis-like mice and spleens and dLNs of MRL/lpr mice (Figures 5A–C). Then, we explored whether the expression of sulforaphane-induced genes was involved in mediating inflammation and autoimmunity. We examined the expression of *Prdx1*, *Nqo1*, *Hmox1*, and *Gss*. The RT-qPCR results showed that *Prdx1* expression was upregulated in the skin lesions of IMQ-induced psoriasis-like mice and in the spleens and dLNs of MRL/lpr mice with sulforaphane administration (Figures 5D,E). These results suggest that sulforaphane may protect against inflammation and autoimmunity in mice *via* upregulating antioxidant gene *Prdx1* expression to reduce the level of oxidative stress.

## Discussion

We found that the administration of sulforaphane ameliorated the IMQ-induced psoriasis-like skin inflammation and renal damage in lupus-prone MRL/lpr mice. In IMQ-induced psoriasis-like mice, sulforaphane reduced the proportions of Th1 and Th17 cells and induced the expression of the antioxidant gene *Prdx1*. Furthermore, our results demonstrated that sulforaphane increased the lifespan and reduced renal damage in lupus-prone MRL/lpr mice, decreased the proportions of plasma cells, Tfh cells, neutrophils, dendritic cells, and the concentration of MDA and induced the expression of antioxidant gene *Prdx1*.

Sulforaphane, which is found in cruciferous vegetables, is an activator of nuclear factor E2-related factor 2 (Nrf2). The antioxidant Nrf2 can suppress lupus nephritis by reducing oxidative stress and the NF-κB signaling pathway (Jiang et al., 2014). Peroxiredoxin 1 (Prdx1) is a member of the peroxiredoxin family that acts as an antioxidant enzyme to catalyze the reduction of hydrogen peroxide (Park et al., 2016). Studies have shown that Prdx1 decreased ROS to protect against oxidative stress (Ding et al., 2017). Quinone oxidoreductase (NQO1), which belongs to the Phase II detoxification enzyme family, catalyzes the two-electron reduction of quinone to the redox-stable hydroquinone (Luo et al., 2019). Nqo1, which exerts antioxidant activity to protect against oxidative stress, is induced by sulforaphane and reduces the neuro-cytotoxicity of DA quinone (Han et al., 2007). Haem oxygenase-1 (Hmox1) and glutathione synthetase (Gss) are also antioxidant enzymes, downstream of the Nrf2 signaling pathway (Lu, 2009; Zhao et al., 2019). We found that Prdx1 expression was upregulated in IMQ-induced psoriasis-like mice and MRL/lpr mice after treatment with sulforaphane, suggesting that sulforaphane exerted the antioxidant effects dependent on the activation of Prdx1.

Psoriasis is a chronic hyperplastic skin disease induced by multiple genetic and environmental factors that affects approximately 0.1–3% of the global population (Boehncke, 2018; Rendon and Schakel, 2019). The abnormally differentiated Th1 and Th17 cells release proinflammatory cytokines, including IFN-γ, IL-17, IL-22, and TNF-α, to increase the cutaneous inflammatory response (Benhadou et al., 2019). Th1 cells increase the production of IL-2, TNF-α, and IFN-γ, participating in the development of psoriasis (Rodriguez-Cerdeira et al., 2019). The enhanced Th1 response increases inflammation by upregulating many cytokines, including IL-1, IL-6, IL-8, IL-12, IL-15, interferon-inducible protein-10, and iNOS, to promote keratinocyte proliferation (Grossman et al., 1989; Xie et al., 1993; Rich and Kupper, 2001; Behnam et al., 2005). IL-17 targets innate immune cells, keratinocytes, and endothelial cells, and is the major effector cytokine that drives the pathogenesis of psoriasis (Blauvelt and Chiricozzi, 2018). In this study, our results demonstrated that sulforaphane reduced the percentages of Th1 and Th17 cells to ameliorate acanthosis and dermal inflammatory cell infiltration in IMQ-induced psoriasis-like skin inflammation. ROS-mediated oxidative stress is involved in numerous signaling pathways related to the inflammatory response, contributing to the progression of psoriasis (Briganti and Picardo, 2003). As an antioxidant therapy, dimethylfumarate (DMF) has been used for the treatment of psoriasis and has shown a high degree of efficacy (Rostami and Mrowietz, 2008). Sulforaphane, as an antioxidant, induces the expression of antioxidant gene *Prdx1*, contributing to the recovery of psoriasis.

SLE is a complicated multifactorial autoimmune disease. The molecular mechanisms remain voluminously unknown. An imbalance in the production and degradation of ROS causes the oxidative modification of DNA, inducing DNA damage (Shah et al., 2014). Dendritic cells recognize the self-antigens and present them to B cells, triggering the autoimmune response and excessive production of autoantibodies (Herrada et al., 2019). Tfh cells assist B cells in inducing autoimmune response, playing an important role in the pathogenesis of SLE (Blanco et al., 2016). The deposition of immune complexes recruits neutrophils, inducing local inflammation and damage (Kaplan, 2011). In our study, the administration of sulforaphane decreased the percentages of plasma cells, Tfh cells, neutrophils, and dendritic cells. Moreover, sulforaphane induced the expression of Prdx1 to protect against oxidative stress. N-acetyl cysteine has been reported to attenuate oxidative stress in SLE, suggesting that the antioxidant therapy might be a promising strategy for the treatment of SLE.

In this study, we found that sulforaphane reduced the proportions of Th1 and Th17 cells and the concentration of MDA. Similarly, sulforaphane decreased the proportions of plasma cells, Tfh cells, neutrophils, and dendritic cells. We also found that the expression of antioxidative gene *Prdx1* was upregulated in the sulforaphane-treated group. It was reported that CYP11A1-derived vitamin D3- and lumisterol-hydroxy derivatives protected primary human keratinocytes against radiation and UVB-induced damage, which is associated with Nrf2-regulated antioxidants responses (Chaiprasongsuk et al., 2019; Slominski et al., 2020). Previous study showed that the oxidative stress and molecular modifications induced by oxidative stress caused the massive activation of immune cells (Lightfoot et al., 2017). We speculated that the activation of NRF2 signaling and reduced oxidative stress contributed to inhibition of activation of immune cells. Moreover, there are other possible mechanisms that sulforaphane ameliorate skin lesion and renal damage. 20(OH)D_3_ and 20,23(OH)_2_D_3_ play as antagonists of RORα and RORγ to inhibit the activity of *IL17* promoter (Slominski et al., 2014). In addition, we suppose that sulforaphane functions as a ligand of transcription factor to inhibit differentiation of immune cells. We conducted the macromolecular docking using the crystal structure of RORγT and sulforaphane. Interestingly, the docking score revealed that sulforaphane might bind to RORγT as a ligand (Supplementary Figure S4 and Supplementary Table 2). We will further study the underlying molecular mechanisms of sulforaphane-induced antioxidation and repression of autoimmune activities in psoriasis and SLE. Moreover, because this condition involves a local inflammatory response, sulforaphane might have a higher degree of efficacy when administered locally in IMQ-induced psoriasis-like skin inflammation.

In conclusion, the results of this study showed that sulforaphane improve the skin lesion of IMQ-induced psoriasis-like mice and the renal damage in lupus-like mice by inhibiting inflammatory and autoimmune responses and oxidative stress. This evidence suggests the potential value of sulforaphane in treating psoriasis and SLE and highlights the use of antioxidant therapy in inflammatory and autoimmune diseases.

## Data Availability

The original contributions presented in the study are included in the article/Supplementary Material, further inquiries can be directed to the corresponding authors.

## Supplementary Material

Supplementary Figure S1 The gating strategy of Th subtypes.

Supplementary Figure S2 The gating strategy of plasma cells.

Supplementary Figure S3 The gating strategy of Tfh cells, neutrophils, and dendritic cells.

Supplementary Figure S4 The docking result of RORγT and sulforaphane.

## References

[B1] Beek C. H. van Reede E. C. (1977). The Nature and Frequency of the Histological Changes Found in Psoriasis Vulgaris. Arch. Dermatol. Res. 257 (3), 255–264. 10.1007/BF00741841 836075

[B2] Behnam S. M. Behnam S. E. Koo J. Y. (2005). Smoking and Psoriasis. Skinmed 4 (3), 174–176. 10.1111/j.1540-9740.2005.03716.x 15891254

[B3] Benhadou F. Mintoff D. Del Marmol V. (2019). Psoriasis: Keratinocytes or Immune Cells - Which Is the Trigger? Dermatology 235 (2), 91–100. 10.1159/000495291 30566935

[B4] Blanco P. Ueno H. Schmitt N. (2016). T Follicular Helper (Tfh) Cells in Lupus: Activation and Involvement in SLE Pathogenesis. Eur. J. Immunol. 46 (2), 281–290. 10.1002/eji.201545760 26614103

[B5] Blauvelt A. Chiricozzi A. (2018). The Immunologic Role of IL-17 in Psoriasis and Psoriatic Arthritis Pathogenesis. Clin. Rev. Allergy Immunol. 55 (3), 379–390. 10.1007/s12016-018-8702-3 30109481 PMC6244934

[B6] Boehncke W. H. (2018). Systemic Inflammation and Cardiovascular Comorbidity in Psoriasis Patients: Causes and Consequences. Front. Immunol. 9, 579. 10.3389/fimmu.2018.00579 29675020 PMC5895645

[B7] Briganti S. Picardo M. (2003). Antioxidant Activity, Lipid Peroxidation and Skin Diseases. What's New. J. Eur. Acad. Dermatol. Venereol. 17 (6), 663–669. 10.1046/j.1468-3083.2003.00751.x 14761133

[B8] Chaiprasongsuk A. Janjetovic Z. Kim T. K. Jarrett S. G. D'Orazio J. A. Holick M. F. (2019). Protective Effects of Novel Derivatives of Vitamin D3 and Lumisterol against UVB-Induced Damage in Human Keratinocytes Involve Activation of Nrf2 and P53 Defense Mechanisms. Redox Biol. 24, 101206. 10.1016/j.redox.2019.101206 31039479 PMC6488822

[B9] Diani M. Altomare G. Reali E. (2015). T Cell Responses in Psoriasis and Psoriatic Arthritis. Autoimmun. Rev. 14 (4), 286–292. 10.1016/j.autrev.2014.11.012 25445403

[B10] Ding C. Fan X. Wu G. (2017). Peroxiredoxin 1 - an Antioxidant Enzyme in Cancer. J. Cel Mol Med 21 (1), 193–202. 10.1111/jcmm.12955 PMC5192802 27653015

[B11] Gabr S. A. Al-Ghadir A. H. (2012). Role of Cellular Oxidative Stress and Cytochrome C in the Pathogenesis of Psoriasis. Arch. Dermatol. Res. 304 (6), 451–457. 10.1007/s00403-012-1230-8 22421888

[B12] Grossman R. M. Krueger J. Yourish D. Granelli-Piperno A. Murphy D. P. May L. T. (1989). Interleukin 6 Is Expressed in High Levels in Psoriatic Skin and Stimulates Proliferation of Cultured Human Keratinocytes. Proc. Natl. Acad. Sci. U S A. 86 (16), 6367–6371. 10.1073/pnas.86.16.6367 2474833 PMC297840

[B13] Han J. M. Lee Y. J. Lee S. Y. Kim E. M. Moon Y. Kim H. W. (2007). Protective Effect of Sulforaphane against Dopaminergic Cell Death. J. Pharmacol. Exp. Ther. 321 (1), 249–256. 10.1124/jpet.106.110866 17259450

[B14] Herrada A. A. Escobedo N. Iruretagoyena M. Valenzuela R. A. Burgos P. I. Cuitino L. (2019). Innate Immune Cells' Contribution to Systemic Lupus Erythematosus. Front. Immunol. 10, 772. 10.3389/fimmu.2019.00772 31037070 PMC6476281

[B15] Jiang T. Tian F. Zheng H. Whitman S. A. Lin Y. Zhang Z. (2014). Nrf2 Suppresses Lupus Nephritis through Inhibition of Oxidative Injury and the NF-Κb-Mediated Inflammatory Response. Kidney Int. 85 (2), 333–343. 10.1038/ki.2013.343 24025640 PMC3992978

[B16] Jiang Y. Wang W. Zheng X. Jin H. (2020). Immune Regulation of TNFAIP3 in Psoriasis through its Association with Th1 and Th17 Cell Differentiation and P38 Activation. J. Immunol. Res. 2020, 5980190. 10.1155/2020/5980190 32280718 PMC7114769

[B17] Kadam D. P. Suryakar A. N. Ankush R. D. Kadam C. Y. Deshpande K. H. (2010). Role of Oxidative Stress in Various Stages of Psoriasis. Indian J. Clin. Biochem. 25 (4), 388–392. 10.1007/s12291-010-0043-9 21966111 PMC2994563

[B18] Kagami S. Rizzo H. L. Lee J. J. Koguchi Y. Blauvelt A. (2010). Circulating Th17, Th22, and Th1 Cells Are Increased in Psoriasis. J. Invest. Dermatol. 1305, 1373–1383. 10.1038/jid.2009.399 PMC2892169 20032993

[B19] Kaplan M. J. (2011). Neutrophils in the Pathogenesis and Manifestations of SLE. Nat. Rev. Rheumatol. 7 (12), 691–699. 10.1038/nrrheum.2011.132 21947176 PMC3243068

[B20] Kirmit A. Kader S. Aksoy M. Bal C. Nural C. Aslan O. (2020). Trace Elements and Oxidative Stress Status in Patients with Psoriasis. Postepy Dermatol. Alergol 37 (3), 333–339. 10.5114/ada.2020.94265 32792872 PMC7394161

[B21] Lai R. Xian D. Xiong X. Yang L. Song J. Zhong J. (2018). Proanthocyanidins: Novel Treatment for Psoriasis that Reduces Oxidative Stress and Modulates Th17 and Treg Cells. Redox Rep. 23, 130–135. 10.1080/13510002.2018.1462027 29630472 PMC6748681

[B22] Li Z. Guo H. Li J. Ma T. Zhou S. Zhang Z. (2020). Sulforaphane Prevents Type 2 Diabetes-Induced Nephropathy via AMPK-Mediated Activation of Lipid Metabolic Pathways and Nrf2 Antioxidative Function. Clin. Sci. (Lond) 134, 2469–2487. 10.1042/CS20191088 32940670

[B23] Lightfoot Y. L. Blanco L. P. Kaplan M. J. (2017). Metabolic Abnormalities and Oxidative Stress in Lupus. Curr. Opin. Rheumatol. 29, 442–449. 10.1097/BOR.0000000000000413 28639951 PMC5586499

[B24] Lin X. Huang T. (2016). Oxidative Stress in Psoriasis and Potential Therapeutic Use of Antioxidants. Free Radic. Res. 50, 585–595. 10.3109/10715762.2016.1162301 27098416

[B25] Lu S. C. (2009). Regulation of Glutathione Synthesis. Mol. Aspects Med. 30, 42–59. 10.1016/j.mam.2008.05.005 18601945 PMC2704241

[B26] Luo S. Kang S. S. Wang Z. H. Liu X. Day J. X. Wu Z. (2019). Akt Phosphorylates NQO1 and Triggers its Degradation, Abolishing its Antioxidative Activities in Parkinson's Disease. J. Neurosci. 39, 7291–7305. 10.1523/JNEUROSCI.0625-19.2019 31358653 PMC6759025

[B27] Ma N. Zhang Z. Liao F. Jiang T. Tu Y. (2020). The Birth of Artemisinin. Pharmacol. Ther. 216, 107658. 10.1016/j.pharmthera.2020.107658 32777330

[B28] Nickoloff B. J. Xin H. Nestle F. O. Qin J. Z. (2007). The Cytokine and Chemokine Network in Psoriasis. Clin. Dermatol. 25, 568–573. 10.1016/j.clindermatol.2007.08.011 18021894

[B29] Park M. H. Jo M. Kim Y. R. Lee C. K. Hong J. T. (2016). Roles of Peroxiredoxins in Cancer, Neurodegenerative Diseases and Inflammatory Diseases. Pharmacol. Ther. 163, 1–23. 10.1016/j.pharmthera.2016.03.018 27130805 PMC7112520

[B30] Peluso I. Palmery M. (2014). Is a Flavonoid-Rich Diet with Steamer Cooking Safe during Calcineurin Inhibitors Therapy? J. Clin. Pharm. Ther. 39, 471–474. 10.1111/jcpt.12186 24938126

[B31] Perl A. (2013). Oxidative Stress in the Pathology and Treatment of Systemic Lupus Erythematosus. Nat. Rev. Rheumatol. 9, 674–686. 10.1038/nrrheum.2013.147 24100461 PMC4046645

[B32] Raiola A. Errico A. Petruk G. Monti D. M. Barone A. Rigano M. M. (2017). Bioactive Compounds in Brassicaceae Vegetables with a Role in the Prevention of Chronic Diseases. Molecules 23, 1. 10.3390/molecules23010015 PMC5943923 29295478

[B33] Rendon A. Schäkel K. (2019). Psoriasis Pathogenesis and Treatment. Int. J. Mol. Sci. 20, 6. 10.3390/ijms20061475 PMC6471628 30909615

[B34] Rich B. E. Kupper T. S. (2001). Cytokines: IL-20 - a New Effector in Skin Inflammation. Curr. Biol. 11, R531–R534. 10.1016/s0960-9822(01)00312-8 11470428

[B35] Rodríguez-Cerdeira C. Cordeiro-Rodríguez M. Carnero-Gregorio M. López-Barcenas A. Martínez-Herrera E. Fabbrocini G. (2019). Biomarkers of Inflammation in Obesity-Psoriatic Patients. Mediators Inflamm. 2019, 7353420. 10.1155/2019/7353420 31275060 PMC6558610

[B36] Rostami Yazdi M. Mrowietz U. (2008). Fumaric Acid Esters. Clin. Dermatol. 26, 522–526. 10.1016/j.clindermatol.2008.07.001 18755371

[B37] Russo M. Spagnuolo C. Russo G. L. Skalicka-Woźniak K. Daglia M. Sobarzo-Sánchez E. (2018). Nrf2 Targeting by Sulforaphane: A Potential Therapy for Cancer Treatment. Crit. Rev. Food Sci. Nutr. 58, 1391–1405. 10.1080/10408398.2016.1259983 28001083

[B38] Shah D. Mahajan N. Sah S. Nath S. K. Paudyal B. (2014). Oxidative Stress and its Biomarkers in Systemic Lupus Erythematosus. J. Biomed. Sci. 21, 23. 10.1186/1423-0127-21-23 24636579 PMC3995422

[B39] Singh T. P. Zhang H. H. Hwang S. T. Farber J. M. (2019). IL-23- and Imiquimod-Induced Models of Experimental Psoriasis in Mice. Curr. Protoc. Immunol. 125 (1), e71. 10.1002/cpim.71 30615272

[B40] Slominski A. T. Chaiprasongsuk A. Janjetovic Z. Kim T. K. Stefan J. Slominski R. M. (2020). Photoprotective Properties of Vitamin D and Lumisterol Hydroxyderivatives. Cell Biochem Biophys 78 (2), 165–180. 10.1007/s12013-020-00913-6 32441029 PMC7347247

[B41] Slominski A. T. Kim T. K. Takeda Y. Janjetovic Z. Brozyna A. A. Skobowiat C. (2014). RORα and ROR γ Are Expressed in Human Skin and Serve as Receptors for Endogenously Produced Noncalcemic 20-hydroxy- and 20,23-dihydroxyvitamin D. FASEB J. 28 (7), 2775–2789. 10.1096/fj.13-242040 24668754 PMC4062828

[B42] Subedi L. Lee J. H. Yumnam S. Ji E. Kim S. Y. (2019). Anti-Inflammatory Effect of Sulforaphane on LPS-Activated Microglia Potentially through JNK/AP-1/NF-κB Inhibition and Nrf2/HO-1 Activation. Cells 8, 2. 10.3390/cells8020194 PMC6406309 30813369

[B43] Tsokos G. C. Lo M. S. Costa Reis P. Sullivan K. E. (2016). New Insights into the Immunopathogenesis of Systemic Lupus Erythematosus. Nat. Rev. Rheumatol. 12, 716–730. 10.1038/nrrheum.2016.186 27872476

[B44] Watanabe-Fukunaga R. Brannan C. I. Copeland N. G. Jenkins N. A. Nagata S. (1992). Lymphoproliferation Disorder in Mice Explained by Defects in Fas Antigen that Mediates Apoptosis. Nature 356, 314–317. 10.1038/356314a0 1372394

[B45] Wu R. Zeng J. Yuan J. Deng X. Huang Y. Chen L. (2018). MicroRNA-210 Overexpression Promotes Psoriasis-like Inflammation by Inducing Th1 and Th17 Cell Differentiation. J. Clin. Invest. 128 (6), 2551–2568. 10.1172/JCI97426 29757188 PMC5983326

[B46] Xie Q. W. Whisnant R. Nathan C. (1993). Promoter of the Mouse Gene Encoding Calcium-independent Nitric Oxide Synthase Confers Inducibility by Interferon Gamma and Bacterial Lipopolysaccharide. J. Exp. Med. 177, 1779–1784. 10.1084/jem.177.6.1779 7684434 PMC2191051

[B47] Yoh K. Itoh K. Enomoto A. Hirayama A. Yamaguchi N. Kobayashi M. (2001). Nrf2-deficient Female Mice Develop Lupus-like Autoimmune Nephritis. Kidney Int. 60, 1343–1353. 10.1046/j.1523-1755.2001.00939.x 11576348

[B48] Yu Y. Su K. (2013). Neutrophil Extracellular Traps and Systemic Lupus Erythematosus. J. Clin. Cel Immunol 4, 139. 10.4172/2155-9899.1000139 PMC3826916 24244889

[B49] Zhao Y. Sun Y. Wang G. Ge S. Liu H. (2019). Dendrobium Officinale Polysaccharides Protect against MNNG-Induced PLGC in Rats via Activating the NRF2 and Antioxidant Enzymes HO-1 and NQO-1. Oxid Med. Cel Longev 2019, 9310245. 10.1155/2019/9310245 PMC6589278 31281597

