# Supplementary figures and images for: Sulforaphane Ameliorates the Severity of Psoriasis and SLE by Modulating Effector Cells and Reducing Oxidative Stress
Source: Front Pharmacol. 2022 Jan 21;13:805508. doi: 10.3389/fphar.2022.805508 (PMC8814458; doi:10.3389/fphar.2022.805508)

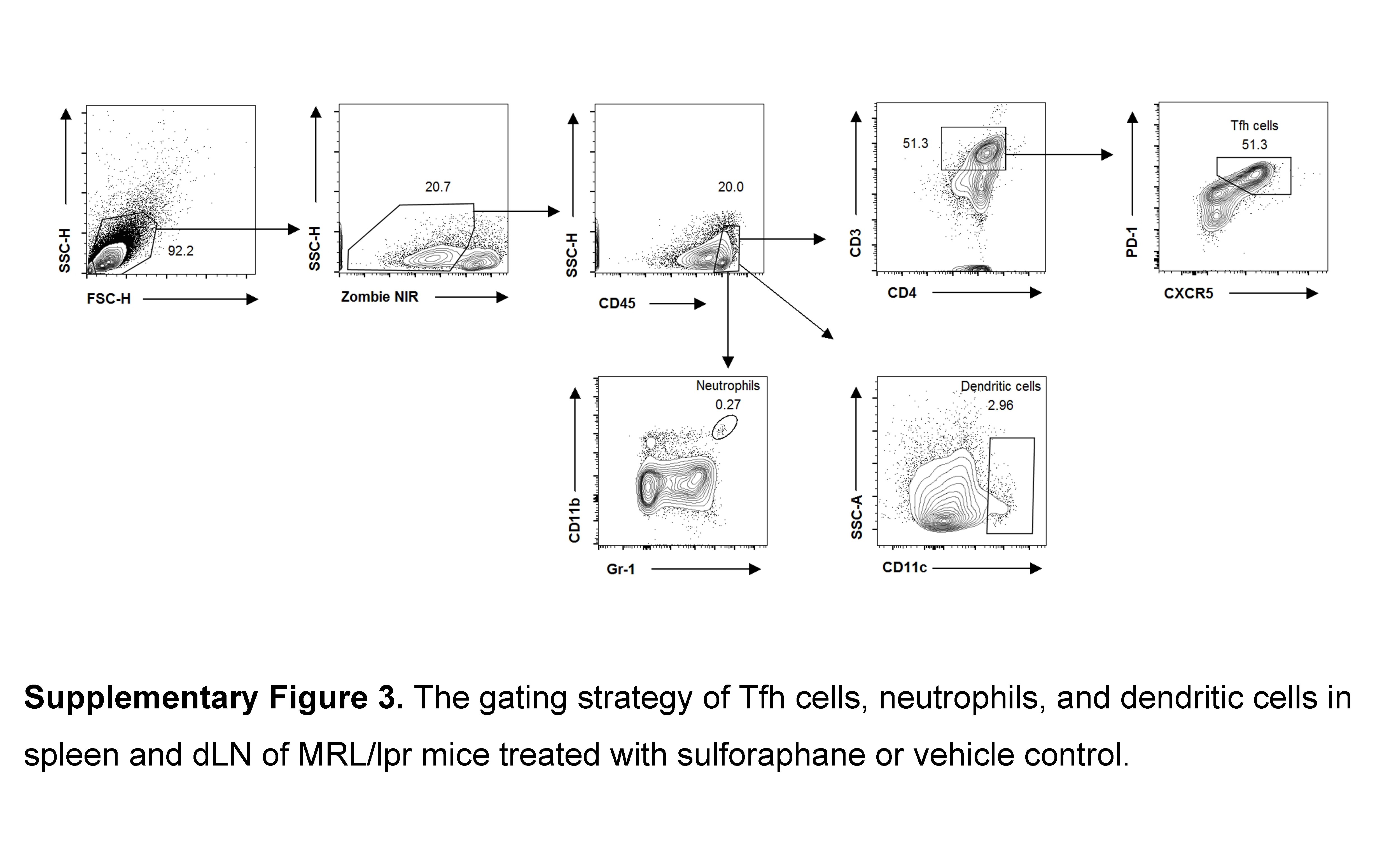

Supplement: Supplementary file 1 [file Image3.JPEG]

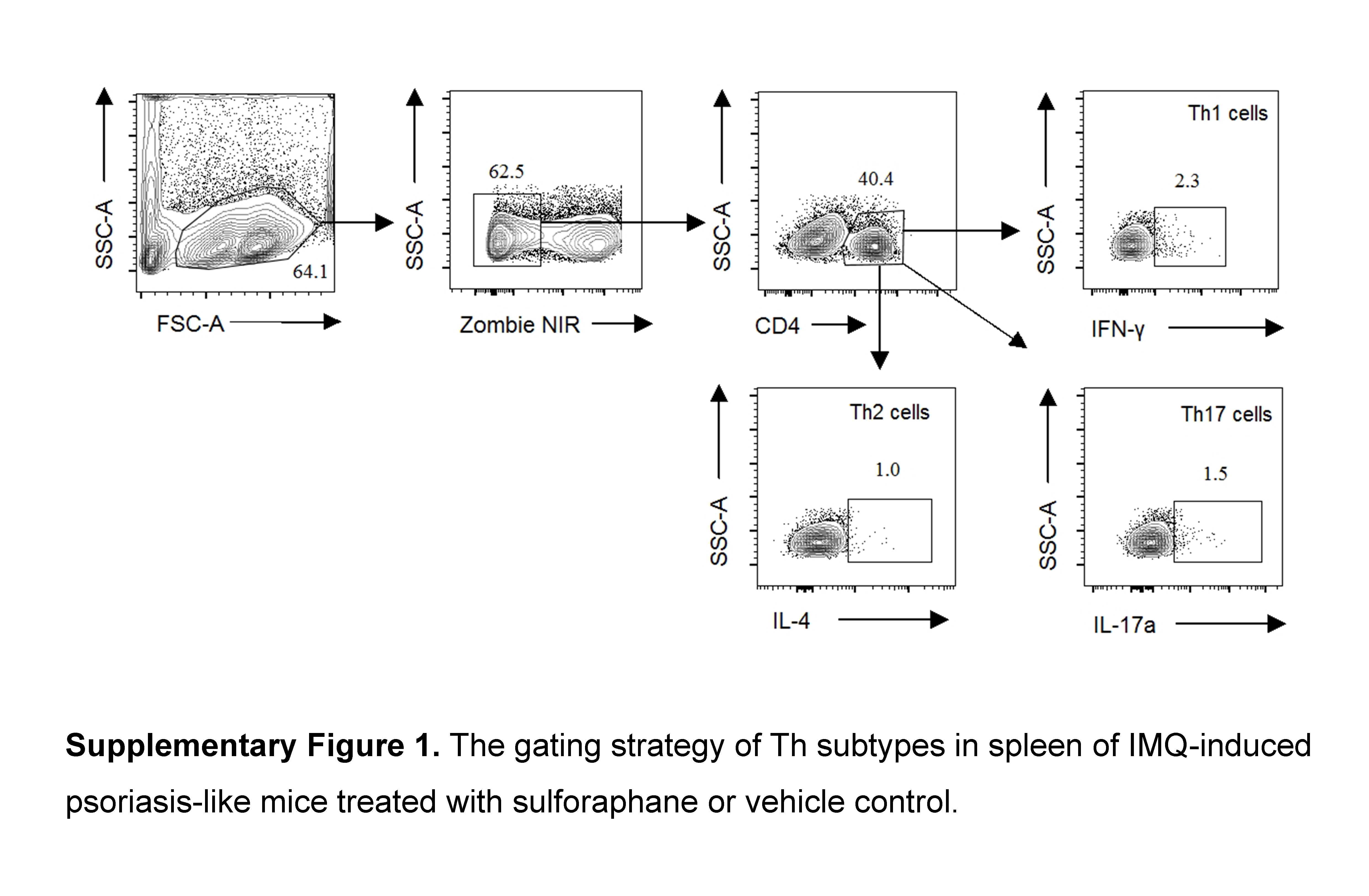

Supplement: Supplementary file 3 [file Image1.JPEG]

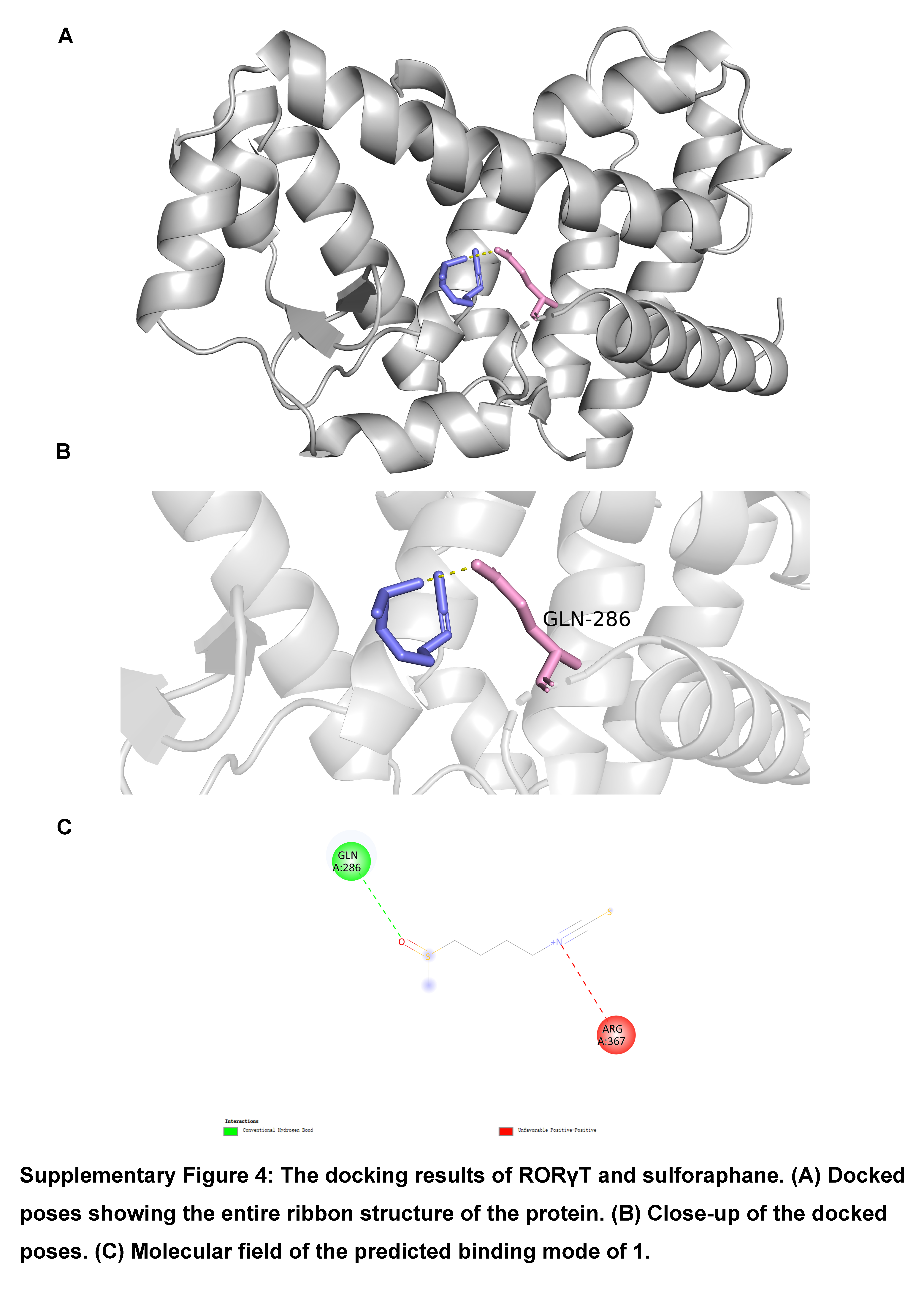

Supplement: Supplementary file 4 [file Image4.JPEG]

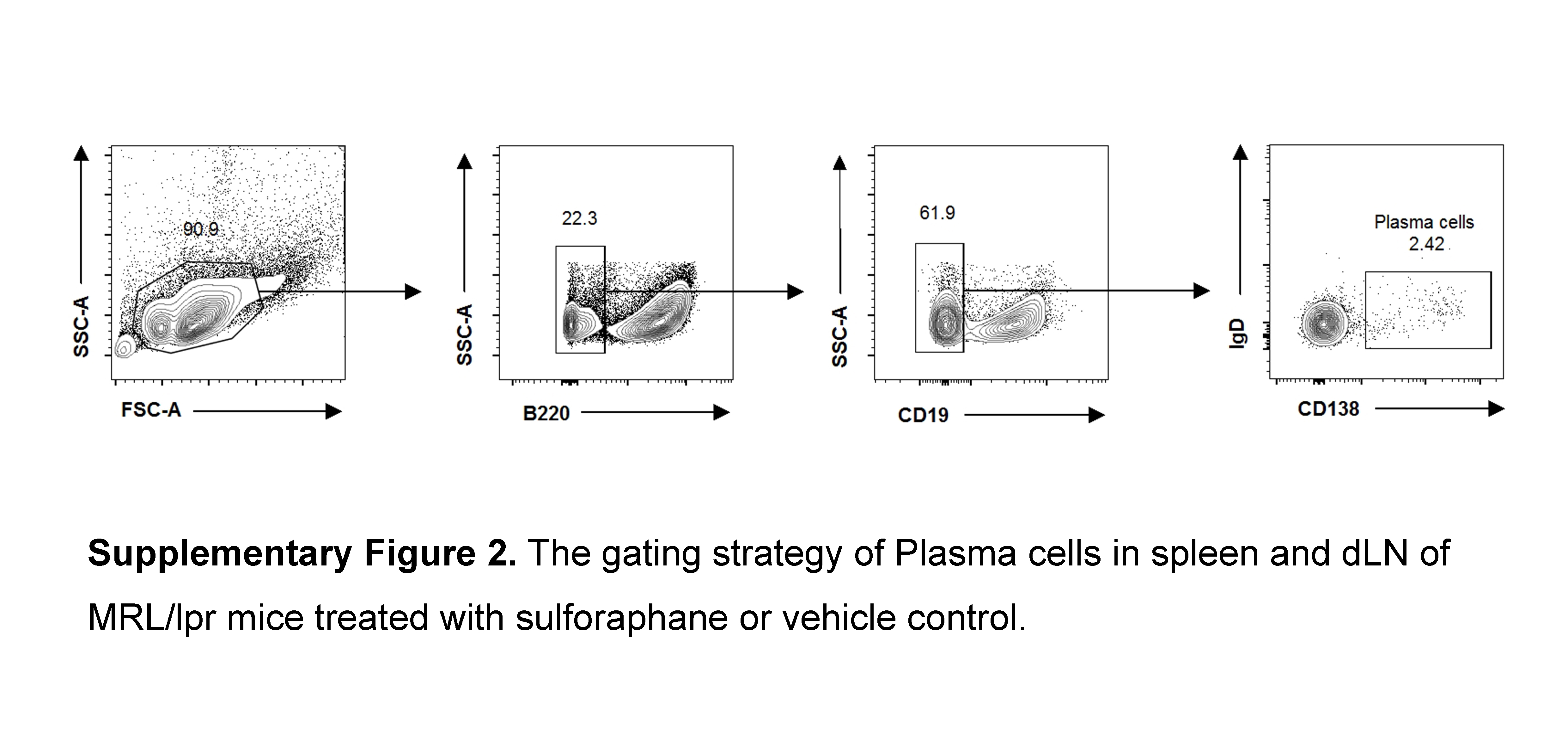

Supplement: Supplementary file 5 [file Image2.JPEG]
