# Supplementary material for: Sulforaphane Ameliorates the Severity of Psoriasis and SLE by Modulating Effector Cells and Reducing Oxidative Stress
Source: Front Pharmacol. 2022 Jan 21;13:805508. doi: 10.3389/fphar.2022.805508 (PMC8814458; doi:10.3389/fphar.2022.805508)
Supplement: Supplementary file 2 [file Table1.DOCX]

**Supplementary Table 1**

**Table S1. Primer sequences**

| Gene | Primer | Sequence (5’–3’) | Application |
| --- | --- | --- | --- |
| *Prdx1* | Forward | TATCAGATCCCAAGCGCACC | RT-qPCR |
|  | Reverse | GGCCAACGGGAAGATCGTTT |  |
| *Gss* | Forward | GATGAACAAGCATGTGGGGC | RT-qPCR |
|  | Reverse | CACACAGGGTAGGGGTTGTC |  |
| *Hmox1* | Forward | GAAATCATCCCTTGCACGCC | RT-qPCR |
|  | Reverse | CCTGAGAGGTCACCCAGGTA |  |
| *Nqo1* | Forward | CATTGCAGTGGTTTGGGGTG | RT-qPCR |
|  | Reverse | TCTGGAAAGGACCGTTGTCG |  |
| *Gapdh* | Forward | TGGAAAGCTGTGGCGTGAT | RT-qPCR |
|  | Reverse | ACACATTGGGGGTAGGAACAC |  |
