# Supplementary material for: Sulforaphane Ameliorates the Severity of Psoriasis and SLE by Modulating Effector Cells and Reducing Oxidative Stress
Source: Front Pharmacol. 2022 Jan 21;13:805508. doi: 10.3389/fphar.2022.805508 (PMC8814458; doi:10.3389/fphar.2022.805508)
Supplement: Supplementary file 6 [file Table2.DOCX]

**Supplementary Table 2**

**Table S2. The docking result of RORγT and sulforaphane**

| Mode | Affinity  (kcal/mol) | Dist from rmsd l.b. | Best mode |
| --- | --- | --- | --- |
| 1 | -3.8 | 0 | 0 |
| 2 | -3.6 | 19.729 | 20.502 |
| 3 | -3.6 | 2.648 | 3.475 |
| 4 | -3.5 | 3.06 | 3.948 |
| 5 | -3.5 | 2.987 | 4.354 |
| 6 | -3.5 | 3.071 | 4.398 |
| 7 | -3.4 | 19.719 | 20.515 |
| 8 | -3.4 | 3.25 | 4.903 |
| 9 | -3.4 | 2.948 | 3.633 |
